# Electrophysiological evidence of RML12 mosquito cell line towards neuronal differentiation by 20-hydroxyecdysdone

**DOI:** 10.1038/s41598-018-28357-2

**Published:** 2018-07-04

**Authors:** Julie Gaburro, Jean-Bernard Duchemin, Prasad N. Paradkar, Saeid Nahavandi, Asim Bhatti

**Affiliations:** 1 Health and Biosecurity, Commonwealth Scientific and Industrial Research Organization, Geelong, VIC Australia; 2 Institute for Intelligent Systems Research and Innovation, Deakin University, Geelong, VIC Australia

## Abstract

Continuous cell lines from insect larval tissues are widely used in different research domains, such as virology, insect immunity, gene expression, and bio pharmacology. Previous study showed that introduction of 20-hydroxyecdysone to *Spodoptera* cell line induced a neuron-like morphology with neurite extensions. Despite some results suggesting potential presence of neuro-receptors, no study so far has shown that these neuron-induced cells were functional. Here, using microelectrode arrays, we showed that the mosquito cell line, RML12, differentiated with 20-hydroxyecdysone, displays spontaneous electrophysiological activity. Results showed that these cells can be stimulated by GABAergic antagonist as well as nicotinic agonist. These results provide new evidence of neuron-like functionality of 20-hydroxyecdysone induced differentiated mosquito cell line. Finally, we used this new model to test the effects of two insecticides, temephos and permethrin. Our analysis revealed significant changes in the spiking activity after the introduction of these insecticides with prolonged effect on the neuronal activity. We believe that this differentiated mosquito neuronal cell model can be used for high-throughput screening of new pesticides on insect nervous system instead of primary neurons or *in vivo* studies.

## Introduction

Neuroactive insecticides remain the principal protection against insects, either to protect crops, livestock or humans from depredation and pathogens transmitted by vectors^1^. The need of functional neurons is very important to identify new compounds and study insecticide effects on the insect nervous system *in vitro*, which is still not well understood. Yet, beyond primary cell culture, which is tedious and time consuming, there is so far no effective technique to provide high number of neurons in a short period of time. Although primary neuron cultures have been developed from a large panel of different insect species at different development stages^2^, this method is not suitable for large scale studies. Insect nervous tissue dissection is very delicate and time-consuming practice^3^.

To overcome this problem, one solution would be to differentiate continuous insect cell lines into functional neuronal networks when needed. Since 1980s, a number of studies have observed that 20-hydroxyecdysone (20HE) in cell culture stimulates neuron-like morphology of cell lines from different species^4–8^. This insect molting hormone stops cell proliferation^9^ and blocks cell division^10^ in various insect cell lines. The interest in this hormone faded until its re-use, a decade later, for its differentiation inducing properties^11,12^. Morphological transformations and induction of long neurite-like extensions by 20HE in the mosquito *Aedes albopictus* C6/36 cells have been reported^13^. Other studies showed efficient coupling effect of insulin/20HE on neurons differentiation of the moth *Spodoptera frugiperda* Sf21 cell line^12,14^.

To overcome this problem, one solution would be to differentiate continuous insect cell lines into functional neuronal networks when needed. Since 1980s, a number of studies have observed that 20-hydroxyecdysone (20HE) in cell culture stimulates neuron-like morphology of cell lines from different species^4–8^. This insect molting hormone stops cell proliferation^9^ and blocks cell division^10^ in various insect cell lines. The interest in this hormone faded until its re-use, a decade later, for its differentiation inducing properties^11,12^. Morphological transformations and induction of long neurite-like extensions by 20HE in the mosquito *Aedes albopictus* C6/36 cells have been reported^13^. Other studies showed efficient coupling effect of insulin/20HE on neuron differentiation of the moth *Spodoptera frugiperda* Sf21 cell line^12,14^.

Although these differentiated cell lines have been characterised morphologically as neuron-like cells, it does not however guarantee neuronal function. Jenson *et al*. showed neuron-like pharmacological properties in Sf21 cell culture treated with 20HE. Caffeine, tetrodotoxin and cobalt antagonists, known ion channel blockers, stopped the hormone induced processes of cell growth and differentiation^12^. Moreover, the use of veratridine, a sodium channel activator^15^, enhances the differentiation and survival effects. These studies highlight potential involvement of neurotransmitter pathways in the differentiation process and suggest a path towards neuronal specialization. In the mosquito *Aedes albopictus* cell line C6/36 treated with 20HE, the authors showed neurite-like long extensions with aggregation of F-actin polymerisation^16^. Combined, these results bring hints that differentiated neuron-like cells could be functionally similar to authentic neuronal cells.

Electrophysiology, defined as the ‘gold standard’ to investigate neuronal signalling^17^, utilises different tools to study neurons from a single ion channel to the activity of hundreds of cells within networks of neurons. The patch-clamp technique is widely used for microscale studies to measure currents of single ion channels; while indirect measurements of large areas of the brain’s activity, such as functional magnetic resonance imaging or electroencephalogram, are used for macroscale studies (*i*.*e*. cm range). However, the patch-clamp technique is limited to only a few neurons per experiment^18^ and macroscale indirect measurements have a low spatial resolution^17^. In order to study neuronal mechanisms at the mesoscale and sample electrophysiological recordings from neuron networks, extracellular recording using microelectrodes is adopted. The metal electrodes are usually integrated to large arrays and called microelectrode array (MEA). This technique enables long-term recordings (from minutes to days) of extra-cellular potentials from a neuron population at a millisecond time scale, with no invasive procedure. There are different MEA chips, described in details, as well as their importance in neuroscience studies, in a review from Obien *et* *al.*^19^. The MEA technology is widely used for neurotoxicology studies^20,21^, mainly in mammalian models^22^. First used with rat primary neuron culture by Pine in 1980^23^, this technology has since been also used in neurophysiology^19^. Work using primary neurons from invertebrates, mainly gastropods such as *Helix*^24,25^, *Aplysia*^26^ and *Lymnaea*^27^ have been reported and reviewed^28^, and so far, for insects, has been only used previously for primary mosquito neurons^29^.

In this study, we differentiated a continuous cell line RML12 from *Aedes albopictus*^30,31^ using 20HE under serum free conditions. After confirming the neuron-like morphology, we used MEA to explore the electrical spiking activity of the differentiated cells. In addition to spontaneous spikes activity, we used chemical stimuli to confirm their neuronal functionality and presence of neurotransmitter receptors. Finally, using multi-well MEA (mwMEA), 20HE differentiated RML12 cells were used for testing effect of insecticides.

## Results

### RML12 cell morphology induced by 20HE treatment

**Figure 1:**
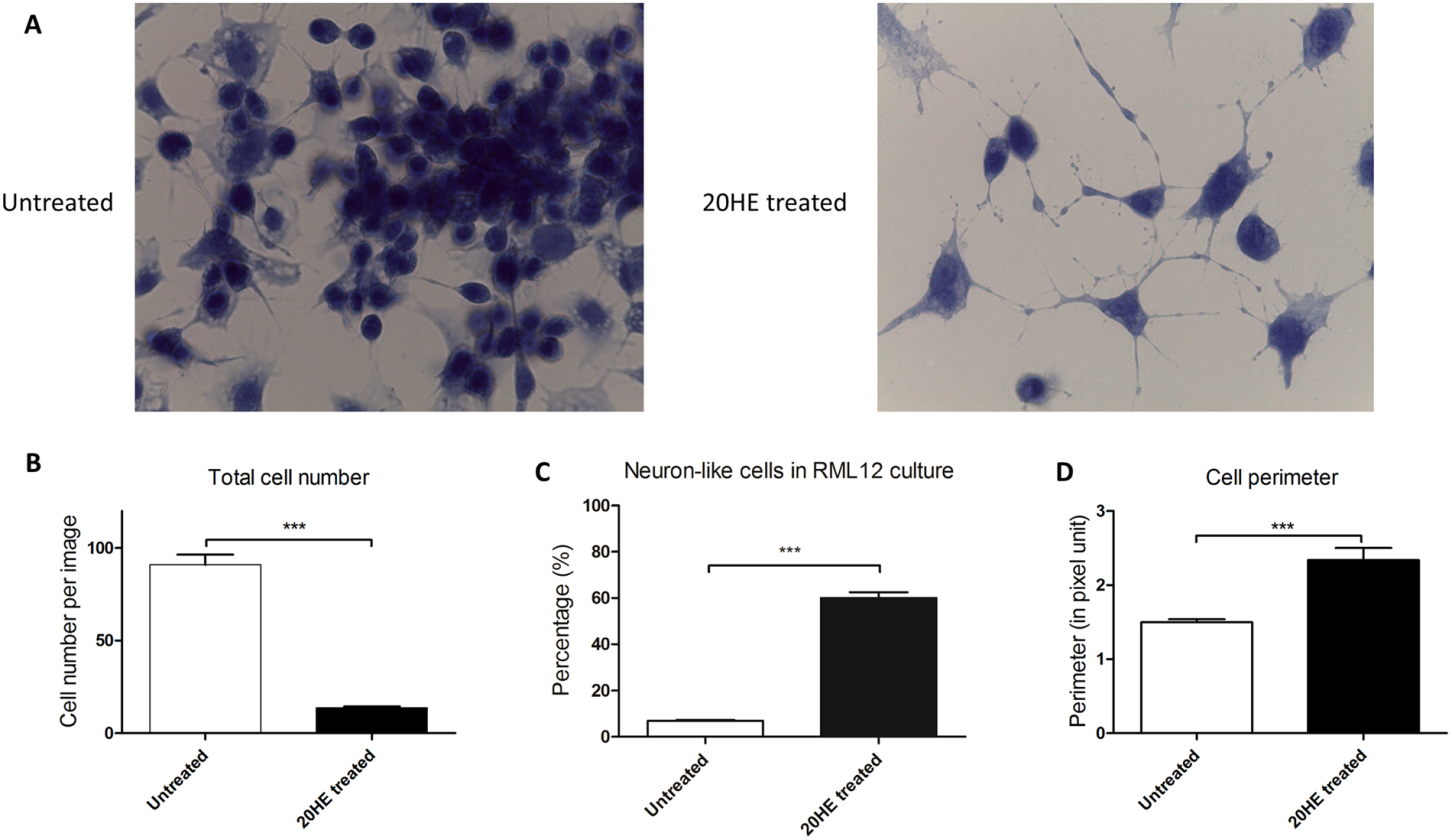
Morphological changes induces by 20-Hydroxyecdysone treatment. (**A**) Images of IHC RML12 cell culture at 5 DIV (magnification × 100). Untreated culture shows numerous small and round clumped cells, whereas 20HE treated culture displays less, neuron-like cells with extensions. With IHC images, different cell parameters, from treated versus untreated cultures, were extracted using ImageJ software. (**B**) Total cell number per image, Mann Whitney test (*N*_untreated_ = 24, *N*_20HE treated_ = 39, *U* = 0.0), *P* < 0.0001. (**C**) Neuron-like cells percentage, with neuron-like defined as cell with at least 3 extensions equal or longer than cell body, Mann Whitney test (*N*_untreated_ = 21, *N*_20HE treated_ = 39, *U* = 0.0), *P* < 0.0001. (**D**) Cell perimeter, Mann Whitney test (*N*_untreated_ = 218, *N*_20HE treated_ = 73, *U* = 4764), *P* < 0.0001.

In this study, we used RML12 cell line from *Aedes albopictus* larvae tissue treated with 2 μg/ml of 20HE in serum free L15 media. To confirm the morphological changes observed after 20HE treatment observed in C6/36^16^ and *Sf21*^12^ cell lines, differentiated cultures were grown on coverslips, fixed and IHC staining was performed (Fig. 1A). At 5 days *in vitro* (DIV), 20HE differentiated cultures showed a significant lower cell number (13.85 on average ± 3.86 sd) than untreated cultures (90.69 on average ± 13.85 sd) (Fig. 1B). Cells extensions, either dendrites or axons, were visible, making the cells asymmetrical. A significant percentage of cells had three or more cell extensions longer than their cell body (Fig. 1C), reaching neighbouring cells like a network. Cells differentiated with 20HE were significantly larger than untreated cells, with a longer cell perimeter, defined as the length of the outside boundary of the cell in pixel unit (cell_20HE treated_ = 2.34 ± 1.4 sd and cell_untreated_ = 1.5 ± 0.57 sd) (Fig. 1D and Supplementary Figure S1).

Time-lapse videos of cell cultures, started at 24 hours post plating, revealed cell culture dynamics. RML12 untreated cells were very motile (Supplementary Video S1), while mosquito primary neuron culture was more stable and showed neuron processes (Supplementary Video S2). RML12 cells differentiated with 20HE and maintained serum-free (Supplementary Video S3) were more motile than the primary neuron culture, however had significantly lower cell travel distance than untreated RML12 cell culture (Table 1).

**Table 1:** Time lapse recording for cell tracking and motility 24 hours post seeding.

Cell culture and condition	Average distance per cell (*N* = 40)	StdDev of distance	*P*, Student’s *T*-test	
RML12, 10% FCS	11.7384	8.1872	*P* = 0.0017	
RML12, serum free/20HE	6.9699	4.2143		*P* = 0.00016
Primary neurons	4.0471	2.0434		

Results are extracted from the ImageJ software with “Manual Tracking” plugin.

### Electrophysiological recording of 20HE differentiated RML12 cultures on microelectrode array

**Figure 2:**
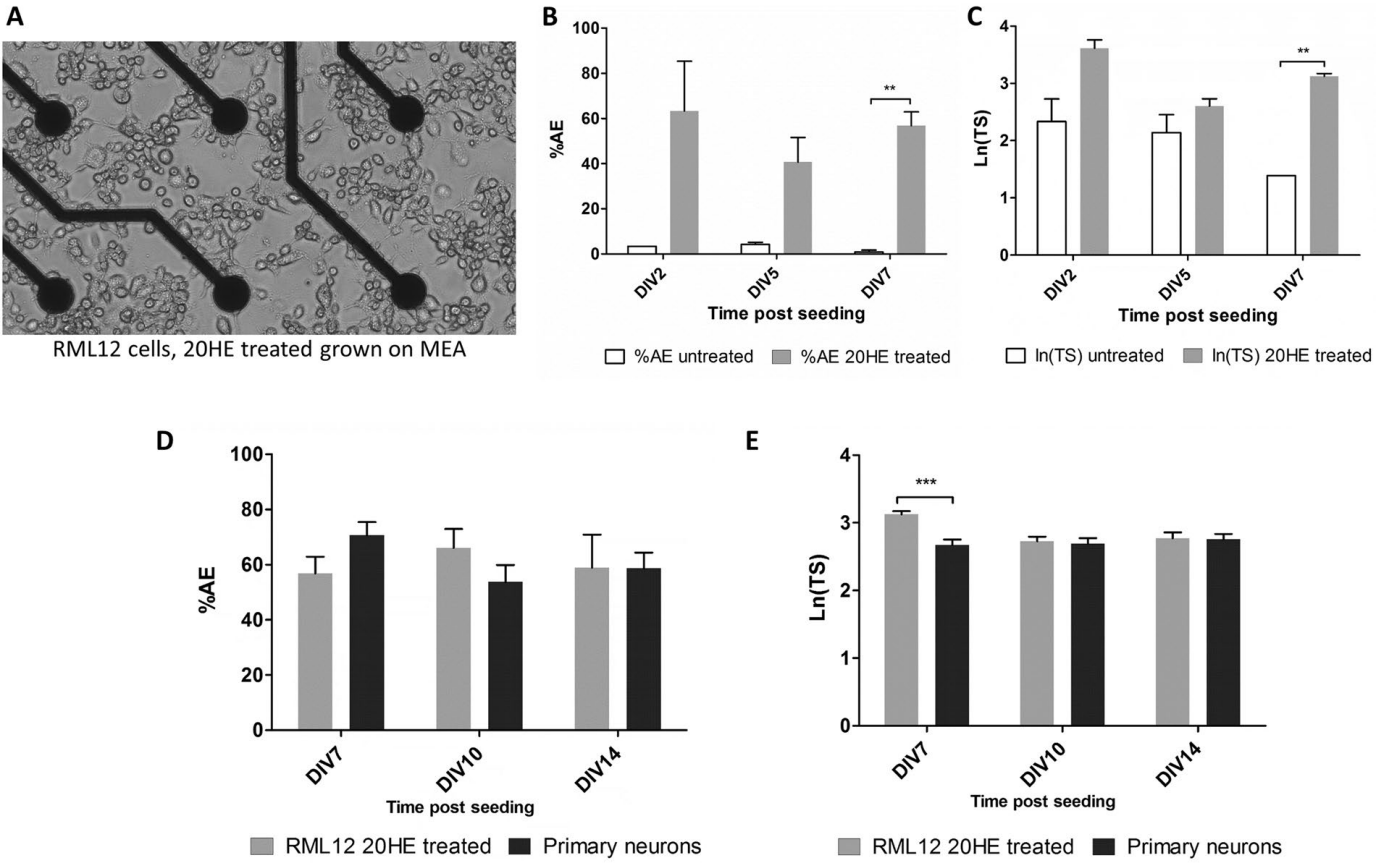
Microelectrode array (MEA) recording and analysis of RML12 cell line treated with 20HE. (**A**) Picture showing RML12 cells 20HE treated cultured on the MEA at 5 DIV (magnification × 20). Recording analysis of RML12 cells untreated (*N* = 3) versus 20HE treated (*N* = 5) from 2 to 7 DIV with (**B**) the percentage of Active Electrodes (AE) and (**C**) the Total Spike (TS) number per AE in natural log (*N*_untreated_ < 5, *N*_20HE treated_ > 100). Bar plots showing the percentage of AE of RML12 20HE treated cultures (*N* = 8) versus *Aedes aegypti* primary neuron cultures (*N* = 7) on MEA from 7 to 14 DIV (**D**), and the ln(TS) number (*N* < 220) of AE from the same cell cultures (**E**).

Although neuron-like processes have been previously observed in 20HE treated insect cell cultures, published work did not confirm yet that cells were electrophysiologically active. After culturing untreated versus 20HE differentiated RML12 cells in MEA wells (Fig. 2A), we recorded the electrical activity of the cell cultures for 10 minutes at various days post plating. Untreated cultures had significantly lower percentage of active electrodes (AE) among the 60 electrodes after 7 DIV (Mann Whitney test, *P* = 0.0078, *U* = 0.0) with less than 2% AE compared to an average of 57% AE for 20HE treated cultures (Fig. 2B). The natural logarithm of total spikes ln(TS) per active electrode were comparable at 2 and 5 DIV in both culture conditions. Untreated cells had a significant lower ln(TS) value after 7 DIV (Mann Whitney test, *P* = 0.0042, *U* = 35.0) (Fig. 2C). Spontaneous activity of 20HE differentiated RML12 cells was compared to the spontaneous spike activity of *Aedes* primary neurons. No significant difference in the percentage of AE at 7, 10 and 14 DIV could be found with an average of 58.9 (±12.02 sem) and 58.6 (±5.8 sem) % AE at 14 DIV for 20HE differentiated RML12 and primary cultures respectively (Fig. 2D). The value of ln(TS) was significantly higher in 20HE differentiated RML12 cultures at 7 DIV (Unpaired *t*-test, *P* < 0.0001, *t* = 5, *df* = 831), however comparable in both types of culture at 10 and 14 DIV (Fig. 2E). Both types of culture also displayed burst activity, defined as episodes of activity (i.e. densely packed spikes) simultaneously occurring at many channels and spread over the entire network^32^. Primary neuron cultures showed burst activity with complex spatial temporal patterns, meaning that more than 3 electrodes were involved in the electrical event, at any time post seeding (Supplementary Figure S2A). 20HE differentiated RML12 cultures showed burst activity with spatial temporal patterns with only 2 electrodes involved at 7 DIV (Supplementary Figure S2B). However, at 10 DIV, bursts started to show more complex spatial temporal patterns, comparable to the ones observed in primary neuron cultures (Supplementary Figure S2C).

**Figure 3:**
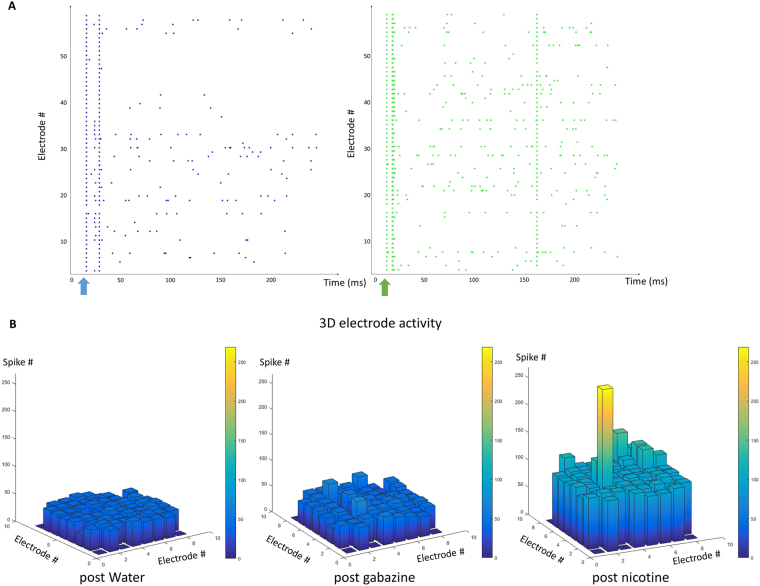
Effect of gabazine and nicotine stimuli on RML12 cell line treated with 20HE and cultured on MEA. (**A**) Raster plots showing the global MEA activity after introduction of either gabazine (left, in blue) or nicotine (right, in green) at 7 DIV. (**B**) Three-dimension (3D) electrode maps showing the effect of gabazine and nicotine on 20HE treated RML12 cells at 7 DIV.

**Figure 4:**
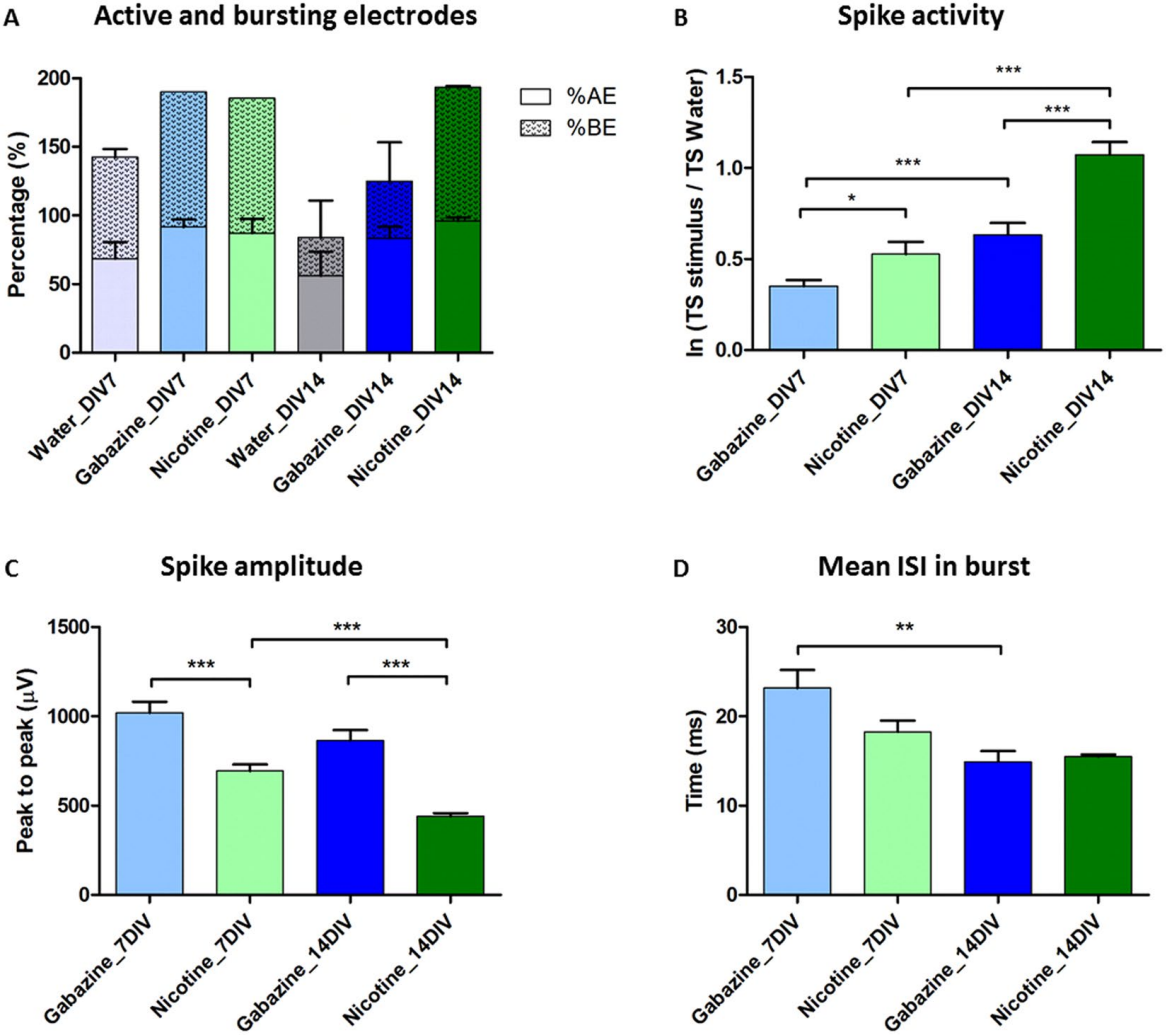
Spiking and bursting parameters of RML12 20HE treated cells after gabazine and nicotine stimuli at 7 and 14 days *in vitro* (DIV). (**A**) Active Electrode (AE) and Bursting Electrode (BE) after the introduction of the solvent (water) or gabazine and nicotine. (**B**) Bar plots showing the natural logarithm ratio of the Total Spike (TS) number from AE after stimulus relative to its solvent (TC-water), with Mann Whitney tests (*P < 0.05; **P < 0.01; ***P < 0.001). (**C**) Spike amplitudes analysis post stimuli of the 20HE treated cells at different DIV, with unpaired *t*-tests (*P < 0.05; **P < 0.01; ***P < 0.001). (**D**) Mean Inter Spike Interval (ISI) in bursts triggered on the network after stimulus (Mann Whitney test for nicotine *N* > 55, *P* = 0.0714, *U* = 2692; Mann Whitney test for gabazine *N* > 50, *P* = 0.0047, *U* = 991.0).

In order to confirm the presence of neurotransmitter receptors in the 20HE differentiated RML12 cultures, nicotine (agonist for acetylcholine nicotinic receptors) or gabazine (antagonist for GABA_A_ receptors) excitatory stimuli were applied at 7 and 14 DIV. The introduction of either gabazine or nicotine triggered an excitatory response of the differentiated neuronal network (Fig. 3A,B). At the network level, we observed an increase in percentage of AE and bursting electrodes (BE) (Fig. 4A). The TS number after the stimuli was compared to the one by solvent as control (Fig. 4B) with the ratio ln(TS_post solvent_/TS_post stimulus_) and the response to each chemical in differentiated cells was stronger (higher TS number) at 14 than 7 DIV. At both times, the response for nicotine was stronger than gabazine. Spike amplitudes were, however, higher after gabazine stimuli than nicotine and decreased with time with significant lower amplitude at 14 DIV than 7 DIV for the nicotine stimulus (Fig. 4C). Finally, a burst parameter known as inter spike interval (ISI), corresponding to the time (in milliseconds) between spikes, was significantly decreased between 7 and 14 DIV for the gabazine stimulus (Fig. 4D).

### Insecticide effect on 20HE differentiated RML12 cultures on multi-well MEA

**Figure 5:**
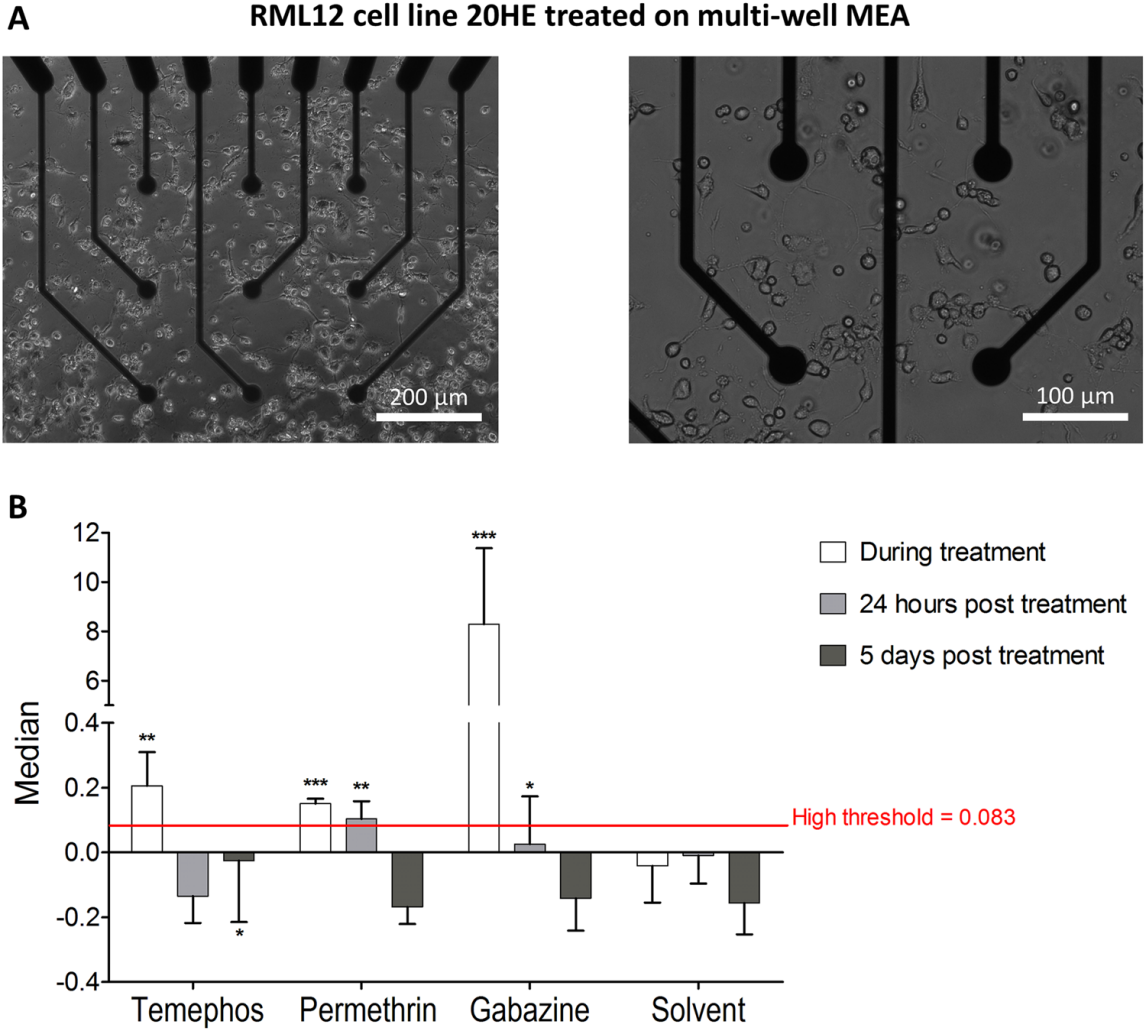
Effect of insecticides on 20HE treated RML12 cell line electrical activity. (**A**) Pictures of RML12 20HE treated cells at 10 DIV on multi-wells MEAat different scales. (**B**) Each bar plot represents the average median of normalized mean firing rate (nMFR) for each group (±sem), directly during treatment (white), 24 hours post treatment (light grey) and 5 days post treatment (dark grey). The high threshold in red was calculated with the median absolute deviation (MAD) of wells treated with solvent. Each condition is made in triplicates (3 separated wells) with 9 microelectrodes, so *N* = 21 for each bar plot. ***Indicates that 3 medians out of 3 wells were higher than the MAD threshold, **indicates that 2 medians out of 3 wells were higher and *only 1 median out of 3 wells was higher than the MAD threshold.

To test if the neuron differentiated cell model could be a potential tool for insecticide study, 20HE treated RML12 cells were cultured on mwMEA for 10 DIV (Fig. 5A). The effect of temephos and permethrin on the cultures were analysed by comparing the median of the normalizing the mean firing rate (nMFR, see methods section for the formula) to the median absolute deviation (MAD) threshold of the control wells where only solvent was introduced. Temephos, an organophosphate product, largely used as larvicide for mosquito control^33^, phosphorylates the acetylcholinesterase enzyme, thus inhibites its ability to hydrolyse acetylcholine and limit its action at the synapse. Permethrin belongs to the pyrethroid insecticide family^1^, which prevents the closure of voltage-gated sodium channels in axons, being excitotoxic. Gabazine was used as positive control. Results showed that both insecticides had an impact on electrical activity of 20HE differentiated RML12 cell cultures. During recording, directly after the introduction of the chemicals, all wells containing permethrin had a nMFR median higher than the threshold, and two out of three wells treated with temephos had a higher nMFR median than the threshold (Fig. 5B). The effect of permethrin can even be observed 24 hours after the introduction of the insecticide with two out of three wells still having a higher nMFR median than the threshold. Five days post insecticide introduction, nMFR was comparable to the one of the wells treated with solvent, except with one temephos well, which had nMFR median higher than the threshold. The percentage of AE pre and post introduction of insecticides were not affected.

## Discussion

**Figure 6:**
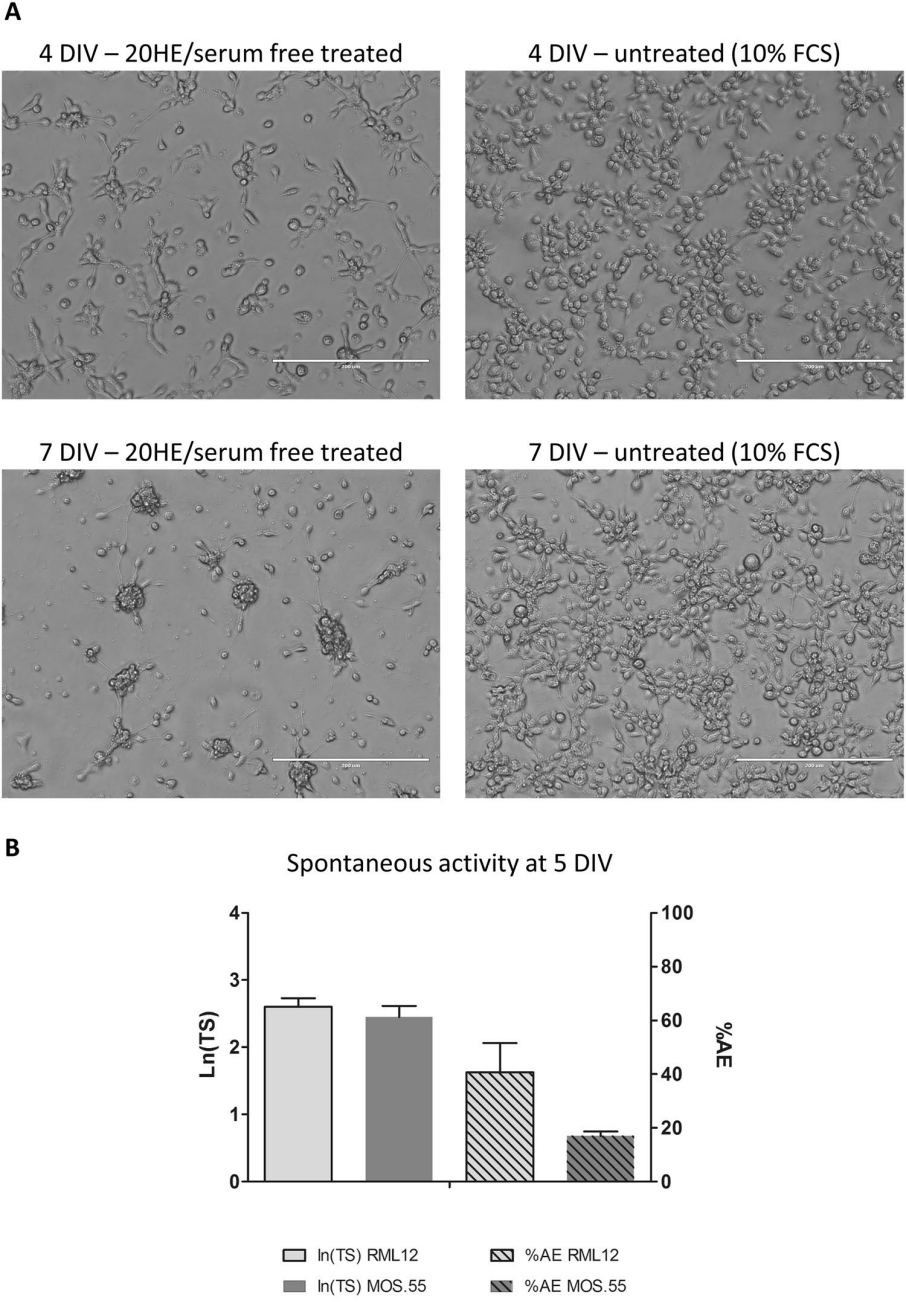
MOS.55 *Anopheles* continuous cell line treated with 20HE and compared to treated RML12 cell cultures. (**A**) MOS.55 20HE treated/serum free (left) *versus* untreated cell lines at 4 and 7 days post seeding, scale bars correspond to 200 µm. (**B**) Recording of spontaneous activity of MOS.55 at 5 div and compared to 20HE treated RML12 cultures: left y-axis shows the average number of total spikes (ln(TS)) and the right y-axis the percentage of active electrodes (AE).

In this paper, we report that the *Aedes albopictus* RML12 cell line, derived from whole mosquito larvae, is responsive to the insect moulting hormone, 20HE, differentiating into neuron-like cells and with inhibition of cell proliferation (Fig. 1A). As previously shown, these observations are typical of ecdysteroid activity on other cell lines, from fly^34,35^, moth^12,36^, mosquito^13,16^ and other insects^37^. The same morphological changes were also observed in *Anopheles* MOS.55 cell lines (Fig. 6A).

Although reaching a neuronal morphology after ecdysteroid or agonist treatments is well documented, electrophysiological properties of these cultures were unknown. The rising phase of action potential is created by voltage-gated sodium channels and necessary for the generation of electric signal in most excitable cells^38^. Some indirect evidence of the presence of voltage-gated sodium channels in 20HE treated Sf21 cultures was shown by veratridine treatment, a sodium channel activator^15^, increasing the differentiation and survival of the cells. In rat brain primary neuron cultures veratridine had similar effects on neuron survival^39^, suggesting that the differentiated Sf21 cells respond to veratridine in a manner similar to that of authentic neural cells. Neuron-like differentiations are also blocked by TTX, which is highly specific to sodium channels^40^. These evidences underline the involvement of sodium channel in the process of differentiation.

Our model of 20HE differentiated RML12 cells cultured on MEA, is to our knowledge the first proof of spontaneous electrical activity of induced neuron-like insect cells. Our results showed that the process is not limited to the RML12 cells but also *Anopheles* mosquito cell line MOS.55, which treated by 20HE showed comparable spontaneous activity at 5 DIV (Fig. 6B). Untreated RML12 cells showed some restricted electrical activity, only on three or less microelectrodes over 60 (Fig. 2B) per well. Our results suggest that 20HE induced mosquito continuous cell lines are electrically functional, when compared to the electrical activity on MEA of mosquito primary neurons. After a week post seeding, differentiated RML12 cells had a higher spiking activity than *Aedes* primary neurons, which stabilized afterwards to the same rate. This spiking behaviour has also been observed in rat cortical neurons with an increase of spiking rate till 21 DIV, followed by a decrease of spike rate and stabilization of the neuron network after 28 DIV^32^. The presence of network bursts (Supplementary Figure S2), which were comparable to the ones observed in *Aedes aegypti* primary neurons cultures, confirmed a functional neuron network in the differentiated RML12 cultures. Our recording after introduction of gabazine and nicotine indicated the presence of GABA_A_ and acetylcholine nicotinic receptors with higher spiking rates of the differentiated cultures. These responses increased with the maturation of the network (Fig. 4B). The presence of functional acetylcholine receptor and pathway, including acetylcholinesterase, was confirmed by the functional assay with temephos. The presence of voltage gated sodium channels, as suspected by the veratidine experiments^15^ was verified by the effect of pyrethroid insecticide on the culture. Expression profile, using the RT-qPCR technique (Supplementary method), for six genes (Supplementary Table S1) highly expressed in the mosquito’s brain^41,42^, showed an overexpression after 20HE induction compared with untreated RML12 cells (Supplementary Figure S3). Half of the selected genes expressed a higher 2^−ΔΔCt^ value at 6 hours *in vitro* (hiv) after 20HE induction. Two other genes had a higher expression at 48 hiv and one at 24 hiv. These results suggest that an early induction (within the first 48 hours post 20HE induction) of neuron specific genes in the observed neuron-like cells, which could also explain the electrical activity observed at 2 div (Fig. 2B,C).

Insect cell lines responding to 20HE have been proposed for *in vitro* screening and identifying hormone analogues^43,44^. Mosquito vectors, responsible for transmitting harmful pathogens to humans^45^, have valuable cell lines for testing new and chemically diverse insecticides. Our results showed that several mosquito cell lines are responsive to 20HE, hence could be valuable for *in vitro* screening of new substances with an ecdysone-like mode of action. Culturing 20HE differentiated mosquito cell lines on MEA brings this tool a step further into insecticide testing. Neuron differentiated RML12 cells could indeed show spiking activity modifications after insecticide introduction in the culture (Fig. 5B). This new model and technique could open a new way to test synthetic insecticides which have an effect on the insect nervous system^1^, such as organochlorides, organophosphates, pyrethroids^46^, and neonicotinoids^47^. The need of new insecticides is an important issue, as the massive use of synthetic insecticides has caused many species of arthropod pests, including human disease vectors, to develop resistance mechanisms (reviewed in^48^) to withstand insecticide treatments because of the selection pressure on insect populations. Beside toxicology studies, the impact of neurotropic virus could also be assessed by MEA on neuron induced cells and could prove to be an easier method for studying virus/mosquito interactions at the neuronal level^29^.

In conclusion, by using MEA technology, our work validates that 20HE induced cell lines do not only have neuron-like morphology but are also electrophysiologically active. The culture of differentiated RML12 cells on the chips also showed indirect evidence of the presence of GABA_A_ and cholinergic receptors, as well as voltage–gated sodium channels within the 20HE treated cultures. This study brings new tools, for insect *in vitro* studies, to investigate mode of action and resistance mechanisms of insecticides.

## Material and Methods

### RML12 cell line differentiation and neuron-like parameters evaluation

During regular cell line passage, RML12 cells were plated at 5.10^5^ cells/ml on glass coverslips into a 24-well plate. Cultures were either supplemented with L15 medium (10% foetal calf serum, 10% Tryptose Phosphate Broth, fungizone and penicillin-streptomycin at 50 units/ml) or with serum free and 20HE enriched (2 μg/ml in 70% ethanol) medium. Each condition had 6 coverslips replicates. Media was changed every 3–4 days and cells were allowed to grow on coverslips for 5 days. At 5 days *in vitro* (DIV), coverslips were fixed with 4% paraformaldehyde for 40 minutes and rinsed 3 times for 5 minutes with PBS. Cells were then stained by immunohistochemistry (IHC) and images of slides were taken using a Leica DM500 inverted microscope and a Leica digital camera (magnifications ×40). For each coverslips, at least 5 pictures were randomly taken and for each condition, 6 different coverslips were assessed. Images were then analysed with ImageJ software: the total cell number and neuron-like cells were determined. A cell was considered as “neuron-like” if it possessed at least three cytoplasmic extensions longer than its cell body. The cell perimeters (calculated by the software from the values of the pixels along the line) were extracted after applying a standard threshold to the image and applying the “Measure” option in the “Analyse” tab. Time lapse videos of RML12 cell line under different conditions and mosquito primary neuron cultures were made with the ImageJ software by importing single pictures taken every minutes for about 3 days and started 24 hours post cells seeding. Individual cell tracking and distances were calculated using the “Manual Tracking” plugin of the software. In total, for each time lapse video, 40 cells were tracked and their travel distance calculated for 3,900 frames.

### Primary neuron cultures preparation

Adult *Aedes aegypti* mosquito female brains were dissected, then isolated in regular cell culture L15 media. About fifteen to twenty brains were clustered and dissociated by repetitive trituration. Before plating, cells were centrifuged 2 times (5 × g for 3 min) and then resuspended in 50 µl of clean media.

### Cell cultures on Microelectrode Array and electrical activity recording

Prior to cell seeding, MEA (MultiChannel Systems, Reutlingen, Germany) were pre-coated with 100 µl of polyethyleneimine at room temperature for 30 min, followed by three TC-water rinses and finally laminin (0.02 mg/ml, Sigma L-2020) was added for 20 min at 37 °C and 5% CO_2_. RML12 cells or mosquito primary neurons, were plated in the center of the MEAs at 7 × 10^4^ cells per device and allowed to settle for half an hour in the 28 °C incubator. Once the cells had adhered to the bottom of the MEA well, 1 ml of media was added depending of the culture condition (RML12 untreated versus 20HE treated and primary neurons). Half of the culture medium was changed every 3–4 days excluding the day before recording and cultures on MEAs were maintained at 28 °C.

Spontaneous electrical activity was recorded at various days post seeding or DIV. Recording were amplified by MCS 1060-INV amplifier (Multi-Channel Systems, Germany) and recorded by MC_Rack software (Multi-Channel Systems, Germany) at a 10 kHz sampling rate for 10 minutes after letting the chips rest 5–10 minutes on the adaptor. The spontaneous activity recorded was analysed using different parameters: first the general chip activity with the percentage of AE and BE, and secondly the activity of each AE by the natural logarithm of the TS number (ln(TS)) during the time of recording (Supplementary Information Figures S4 and S5). For chemical stimuli at 7 and 14 DIV, electrical activity from MEA cultures was first recorded for 15 minutes after introduction of the solvent (TC-water), then with either nicotine (Sigma Aldrich) or gabazine (SR-95531, Tocris, Bioscience) at the concentration of 100 µM. Media was changed after each recording session, and replaced with its respective media condition.

### Differentiated RML12 cultures on multi-well microelectrode array and insecticide treatments

To test possible effect of insecticide treatment on differentiated cells, RML12 cells were seeded on multi-well microelectrode array (mwMEA), as described above. The mwMEA (MultiChannel Systems, Reutlingen, Germany) are composed of 6 wells with nine microelectrodes at the bottom. This devices are well adapted for excitotoxicity testing, as more wells provides more replicates per conditions^49^. Cells were allowed to differentiate for nine days with regular media changes. Before insecticide treatments, spontaneous electrical activity of mwMEA were recorded for 30 minutes as baseline control.

Two insecticides were used for this experiment: a pyrethroid, permethrin (Sigma Aldrich) and an organophosphate, temephos (Sigma Aldrich), both at final concentration of 40 μM^50^, dissolved in 70% ethanol. Gabazine was used as positive control and solvent as negative control. At the end, each condition was in triplicate over 2 different mwMEAs. All recordings lasted 30 minutes and were first done before treatment (baseline) and then immediately after treatment. The wells were rinsed and media was replaced. Cell activity was also recorded at 24 hour and 5 days post treatment to monitor any long term modifications.

### Microelectrode array recording analysis

Microelectrode array data analysis was performed with the MC_Rack software (Multi Channel Systems, Reutlingen, Germany) offline with raw data. High-pass filtered with cut off frequency of 200 Hz was used to remove low-frequency local field potentials. Active electrodes were selected employing the criterion: electrode spike rate is equal or higher than 0.01 spike per second. Spikes were extracted using threshold-based detector set to an upward excursion beyond 5.5 times the standard deviation above the peak-peak noise level. The average detection threshold was set at 22 μV. A burst was defined by a maximum spike interval within a burst of no more than 100 ms, a minimum burst duration no less than 10 ms and with a minimum number of spikes within a burst no less than 3^51^. A network burst was counted when two individual bursts are happening at the same time.

For spontaneous activity analysis, the percentage of AE per MEA well was calculated, as well as its total spike (TS) number per AE (in natural logarithm). For the stimulus activity analysis post gabazine or nicotine, the relative TS was calculated as ln(TS_post solvent_/TS_post gabazine or nicotine_) to see the difference of activity between solvent and chemical introduction. Spike amplitudes post 20 seconds stimuli were extracted using MC_Rack software, with 3 ms pre-trigger and post-trigger spike cut out parameters. Mcd filtered MC_Rack files of 20 seconds post stimuli or during a bursting event window, were converted into txt files (MC_Data tool, MultiChannel System, Reutlingen, Germany). For raster plots, 3 dimension (3D) electrode maps and network burst analysis the self-built software NeuroSigX was used. NeuroSigX software uses novel spike sorting and data analysis algorithms as described in^52,53^, to explore the neural spike activity and spatio-temporal behaviour of the neuronal network (http://www.deakin.edu.au/~asimbh). A threshold of 22 µV is employed to maintain the analytical consistency between the preliminary analysis by MC_Rack software and analysis by NeuroSigX.

Spike activity analysis after insecticides treatment was adapted from^50^. Effect of compounds were assessed via effects on network firing rates by normalizing the mean firing rate (nMFR) in each well, with the formula: nMFR = −1 (MFR_treatment_ − MFR_baseline_)/(0 − MFR_baseline_). For wells treated with solvent, median absolute deviation (MAD) of nMFR were determined, and where the nMFR median of insecticide treated wells exceeded twice the MAD of solvent-treated wells (MAD threshold = 0.083), they were considered as abnormal^54^, with significant effect on spontaneous spike activity.

### Statistical analysis and graphics

Bar plots and statistical analysis are done using GraphPad Prism 5 software. All statistical tests are done using a two-tailed analysis and results are expressed with the *p*-value using the following annotations: ns for *P* > 0.05, *for *P* ≤ 0.05, **for *P* ≤ 0.01, and ***for *P* ≤ 0.001. Graphical representation of the raster plots, 3D electrode activity maps and network burst parameters analysis, were extracted from NeuroSig software.

## Electronic supplementary material

Supplementary Information

Supplementary Video S1

Supplementary Video S2

Supplementary Video S3
