# Supplementary material for: Electrophysiological evidence of RML12 mosquito cell line towards neuronal differentiation by 20-hydroxyecdysdone
Source: Sci Rep. 2018 Jul 4;8:10109. doi: 10.1038/s41598-018-28357-2 (PMC6031678; doi:10.1038/s41598-018-28357-2)
Supplement: Supplementary file 1 — Supplementary Information [file 41598_2018_28357_MOESM1_ESM.pdf]

Supplementary Information

Electrophysiological evidence of RML12 mosquito cell line towards neuronal differentiation by 20-hydroxyecdysdone

Julie Gaburro[1, 2 *], Jean-Bernard Duchemin[1 *], Prasad. N Paradkar[1], Saeid Nahavandi[2] and Asim Bhatti[2]

[1] Health and Biosecurity, Commonwealth Scientific and Industrial Research Organization, Geelong, VIC, Australia.

[2] Institute for Intelligent Systems Research and Innovation, Deakin University, Geelong, VIC, Australia,

[*] Corresponding authors

This document includes:

- **Supplementary method** (RT-qPCR) for RML12 RNA expression.

- **Tables**:

    - Table S1: List of 5-3’ sequences of oligonucleotides primers used for RT-qPCR.

- **Figures**:

    - Figure S1: ImageJ image processing.

    - Figure S2: Burst parameter analysis of RML12 20HE treated cells to primary neurons.

    - Figure S3: Comparison of gene expression, via RT-qPCR of RML12 20HE induced versus untreated at different time points.

    - Figure S4: Microelectrode array raw recording and analysis parameters illustration during spontaneous activity from a neuronal network.

    - Figure S5: MC_Rack electrode recording of 20HE induced RML12 culture spontaneous activity.

- **Time-lapse videos**:

    - Video S1

    - Video S2

    - Video S3

SUPPLEMENTARY METHOD

Gene expression (with Quantitative RT-PCR) of RML12 cells during the first week of 20HE induction.

After 6, 24 and 48 hours *in vitro* (hiv) or 7 days *in vitro* (div) RML12 cells untreated and 20HE induced were stored in RLT-buffer at -80 °C before testing. Total RNA was extracted from the samples using RNeasy Plus Mini Kit (Qiagen Sciences, Maryland, MA) and cDNA was prepared using random hexamers and Superscript-III reverse transcriptase (Thermo Fisher Scientific Inc. Australia) as per manufacturer's protocol. Real-time PCR assay was performed using the SYBR® Premix Ex Taq™ II (Takara-Bio Inc, China) on a QuantStudio™ 6 Flex Real Time PCR System (Applied Biosystems). The primers used for gene expression are listed in the table below. Cycling was as follows: 95°C for 30 seconds, followed by 45 cycles of 95°C for 5 seconds, 55°C for 40 seconds, followed by melt-curve analysis. The cycle threshold (Ct) values, in duplicate for each sample, were collected at each time point for each condition. For RNA expression, the $2_{\Delta\Delta Ct}$ values were calculated at each time point for each gene as the fold-increase over untreated control and normalized to Rsp17 housekeeping gene expression at the same time point. Ct values were measured at 6, 24 and 48 hpi and at 7 div at each time and compared to control values of untreated cells at the same time.

The genes coding for the ionotropic histamine-gated chloride channel (HisCl2) and Glutamate decarboxylase 1 (Gad1), are two genes highly expressed in the brain of female *Aedes aegypti* (1). Gad1 is a nervous system-specific glutamic acid decarboxylase, which synthetize the major inhibitory neurotransmitter gamma-Aminobutyric acid (GABA). HisCl2 is required for vision and is essential in the first-order interneurons to receive histaminergic inputs from the photoreceptors. The other genes used for RNA expression, the cAMP-dependent protein kinase A (PKA1), the cAMP-responsive element-binding protein (CREB2), the calcium/calmodulin-dependent protein kinase type II (CAMKii) and synapsin, are genes involved in synaptic plasticity of invertebrate's brain (2).

References:

(1) Matthews, B. J., McBride, C. S., DeGennaro, M., Despo, O., & Vosshall, L. B. (2016). The neurotranscriptome of the *Aedes aegypti* mosquito. *BMC genomics*, 17(1), 32.

(2) Kandel, E. R. (2012). The molecular biology of memory: cAMP, PKA, CRE, CREB-1, CREB-2, and CPEB. *Molecular brain*, 5(1), 14.

SUPPLEMENTARY TABLES

Table 1: List of 5-3’ sequences of oligonucleotides primers for quantification gene expression 20HE induced RML12 cells.

Gene	Forward primer / Reverse primer
**Rsp17 (Housekeeping)**	AACGAAGCCCCTGCGCACAA / CCTGCTCCAGGGCGGACACT
**cAMP-dependent protein kinase catalytic subunit, transcript variant X3 (PKA; LOC5569015)**	GGGGAACAACGCTACATCCA / GGCAGCAGTGTTGGTAGGAT
**calcium/calmodulin-dependent protein kinase type II alpha chain, transcript variant X1 (CAMK II; LOC5578710)**	CACTGCCATCAGAATGGGGT / GGACTTCGATCGCTAGACCG
**cAMP-responsive element-binding protein-like 2, transcript variant X1 (CREB2; LOC5569180)**	ATTGACAGTGGTTGAGGAGCA / TTCTCAGTAGACGCCTTGCG
**Synapsin (LOC5565053)**	GACGTCTCGGAAAGGAAGCA / CCGTAACAGTGACCAGCCTT
**Ionotropic histamine-gated chloride channel (HisCl2; AAEL012248)**	GTCGTAGACGAGCACATCGAG/ CAGGTTGGAATGTACGTGTGG
**Glutamate decarboxylase 1 (Gad1; AAEL011981)**	CACGTATGAGATTGCACCGGTG/ GGTCTTTGTAGCCTGGGAAC

SUPPLEMENTARY FIGURES

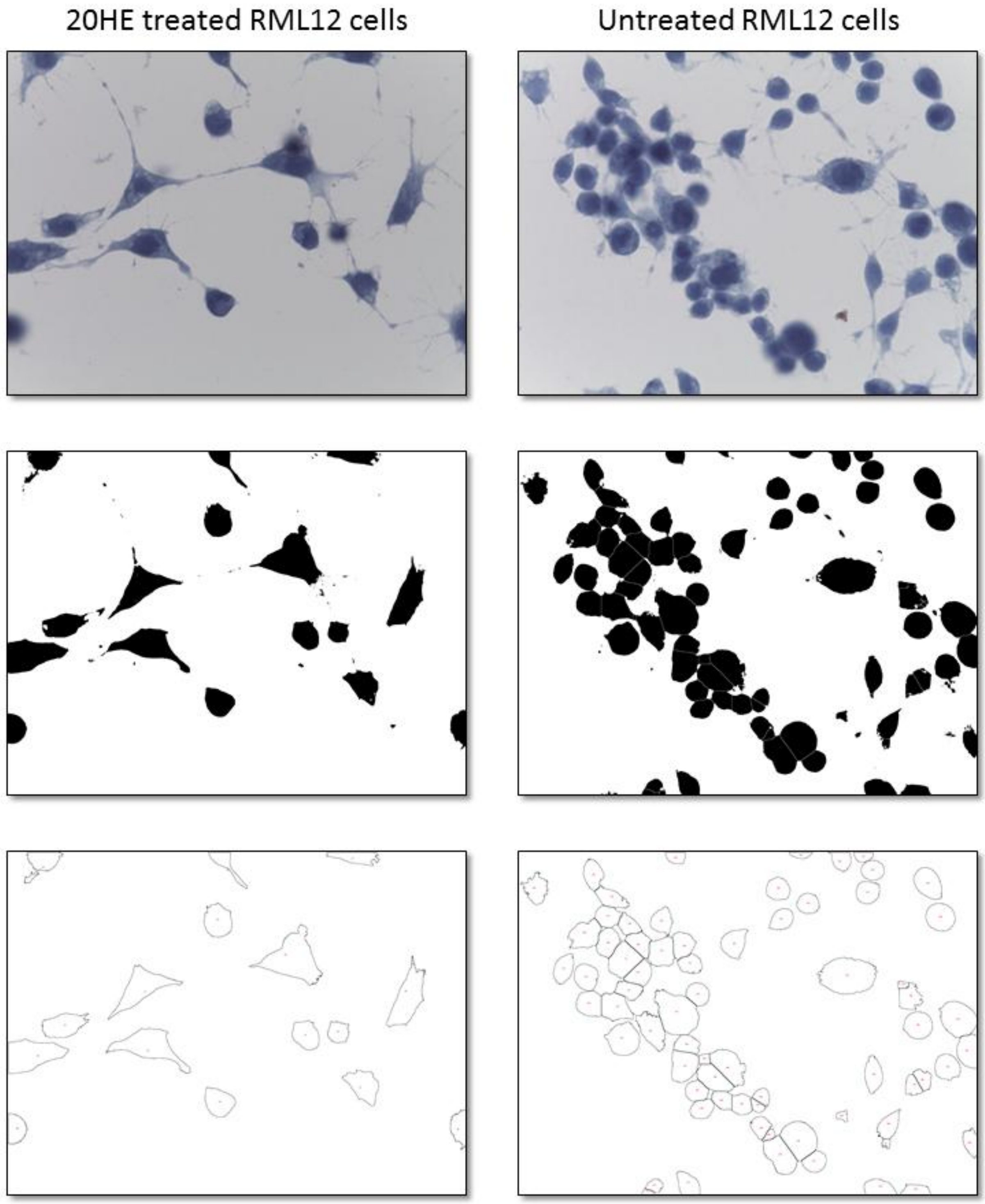

Supplementary Figure S1. ImageJ image processing. Examples of IHC images (top) being converted to 8-bits images and processed with a threshold filter (middle) allowing cell perimeter calculation by the software (bottom).

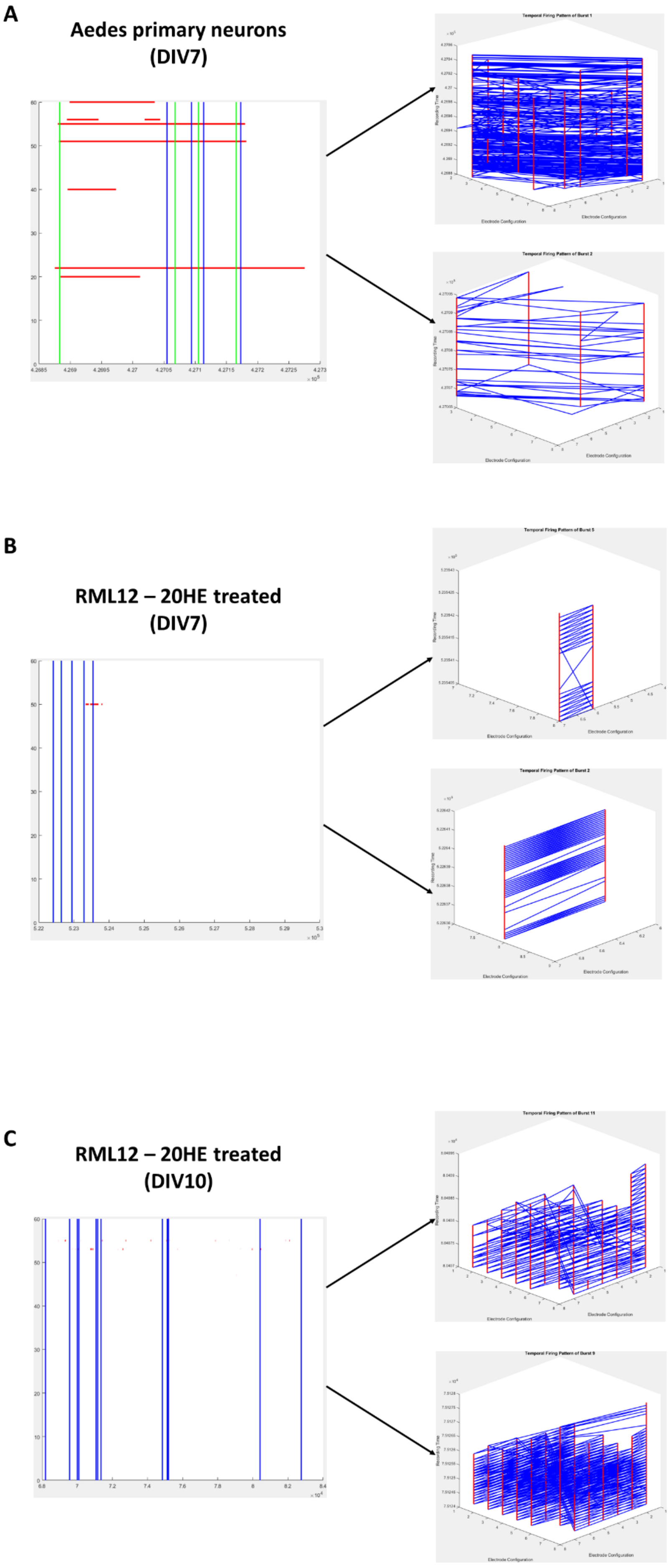

A
Aedes primary neurons
(DIV7)
Temporal Firing Pattern of Burst 1
Temporal Firing Pattern of Burst 2
Recording Time
Electrode Configuration
B
RML12 – 20HE treated
(DIV7)
Temporal Firing Pattern of Burst 5
Temporal Firing Pattern of Burst 2
C
RML12 – 20HE treated
(DIV10)
Temporal Firing Pattern of Burst 11
Temporal Firing Pattern of Burst 9

Supplementary Figure S2. Burst parameter analysis of RML12 20HE treated cells to primary neurons. (**A**) *Aedes aegypti* primary neuron cultures between 7 and 14 DIV show burst activity, with electrode bursts (red lines) and network bursts (green lines for burst start and blue for stop). Examples of burst spatio-temporal pattern from network bursts, show dense and numerous electrodes involved in electrical event. (**B**) Same analysis done with RML12 20HE treated cells at 7 DIV. Network bursts are present but spatio-temporal patterns less dense and only two electrodes involved in most of the network bursts. (**C**) Same analysis done with RML12 20HE treated cells at 10 DIV. Network bursts are this time more complex and similar to primary neuron cultures network bursts, showing a potential maturation of the differentiated network.

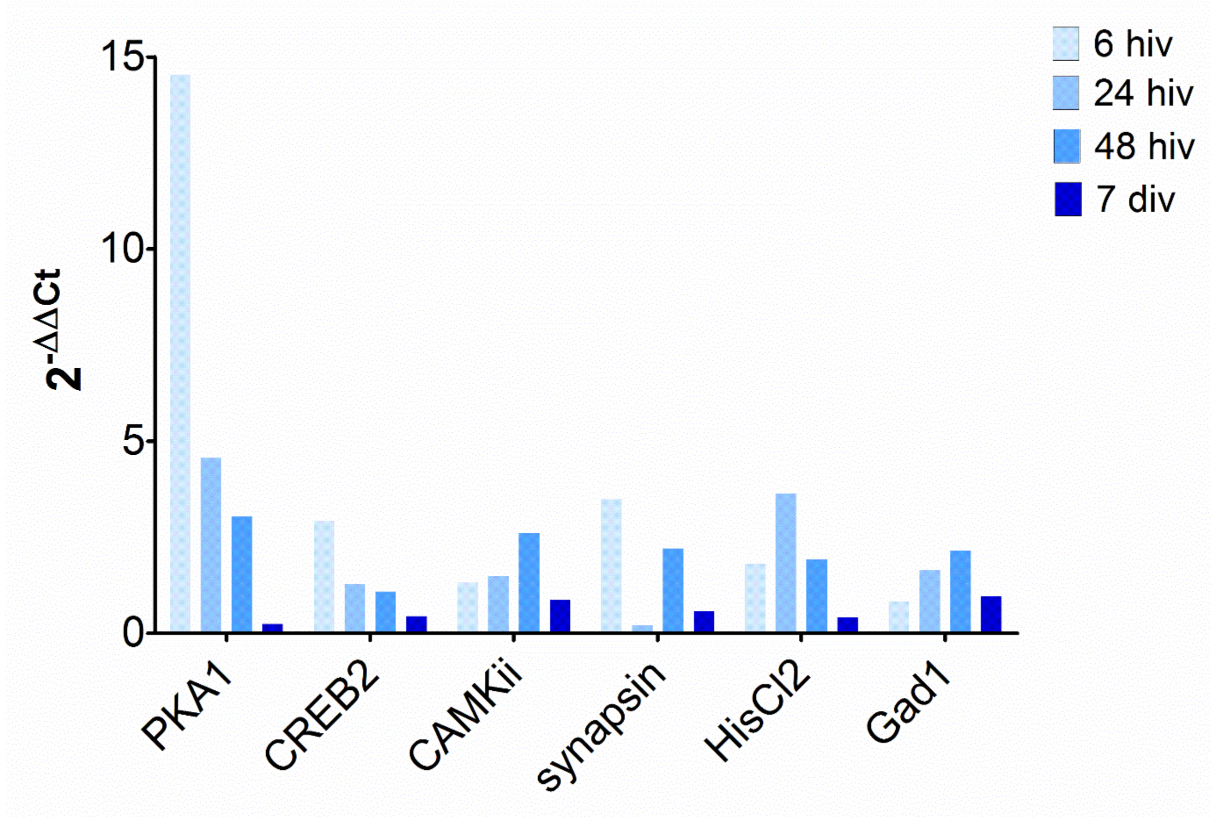

Supplementary Figure S3. Gene expression of RML12 cells 20HE treated at different time points post induction. Means (from duplicates) of RNA expression fold change detected via RT-qPCR in $2_{\Delta\Delta Ct}$ values, compared to Rsp17 housekeeping gene and to untreated cells, for the corresponding day. Gene expression was done with the following genes: cAMP-dependent protein kinase catalytic subunit 1 (PKA1), cAMP-responsive element-binding protein-like 2 (CREB2), calcium/calmodulin-dependent protein kinase type II alpha chain (CAMKii), synapsin, ionotropic histamine-gated chloride channel (HisCl2), and Glutamate decarboxylase 1 (Gad1).

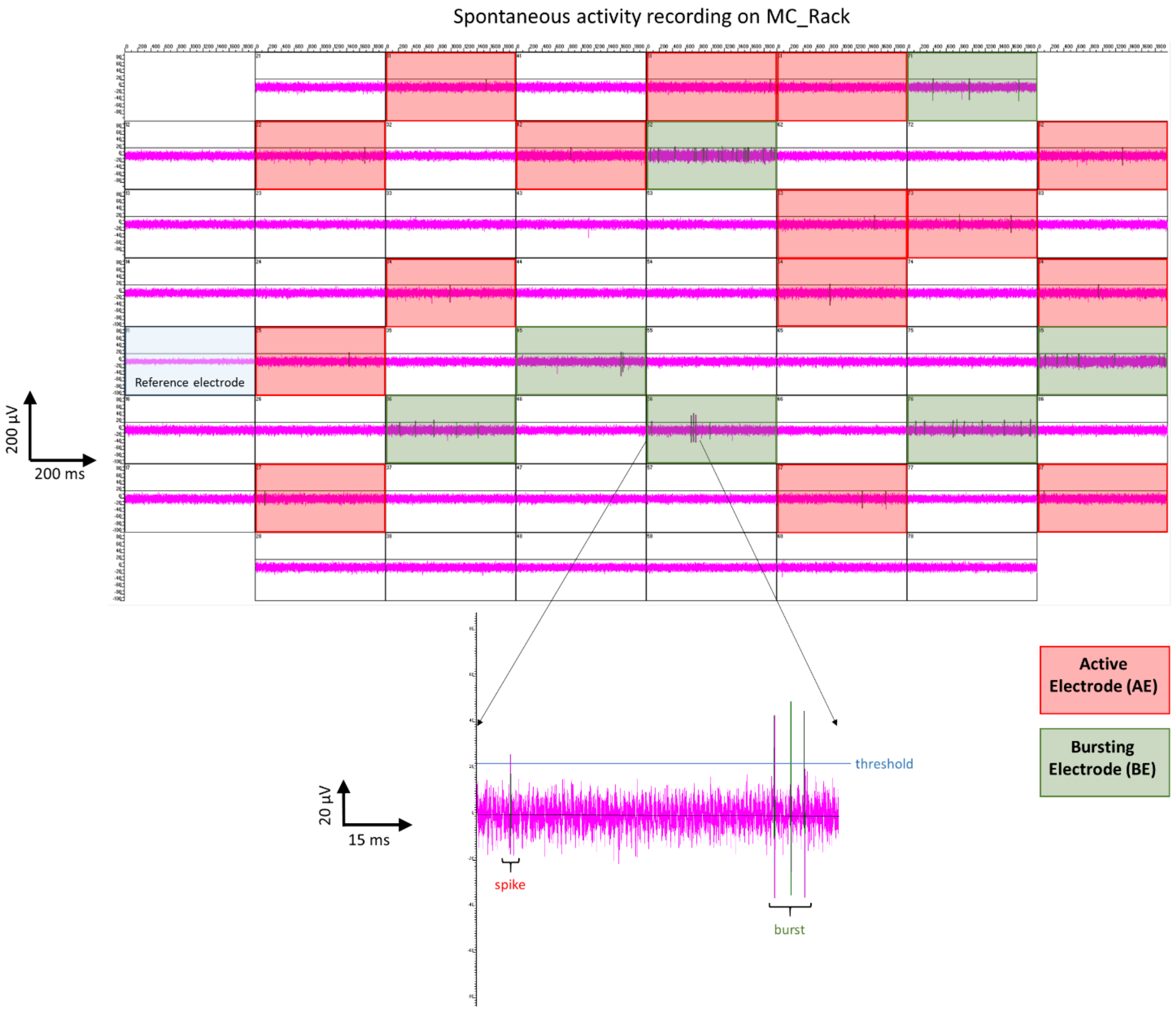

Supplementary Figure S4: Microelectrode array raw recording and analysis parameters illustration during spontaneous activity from a neuronal network.

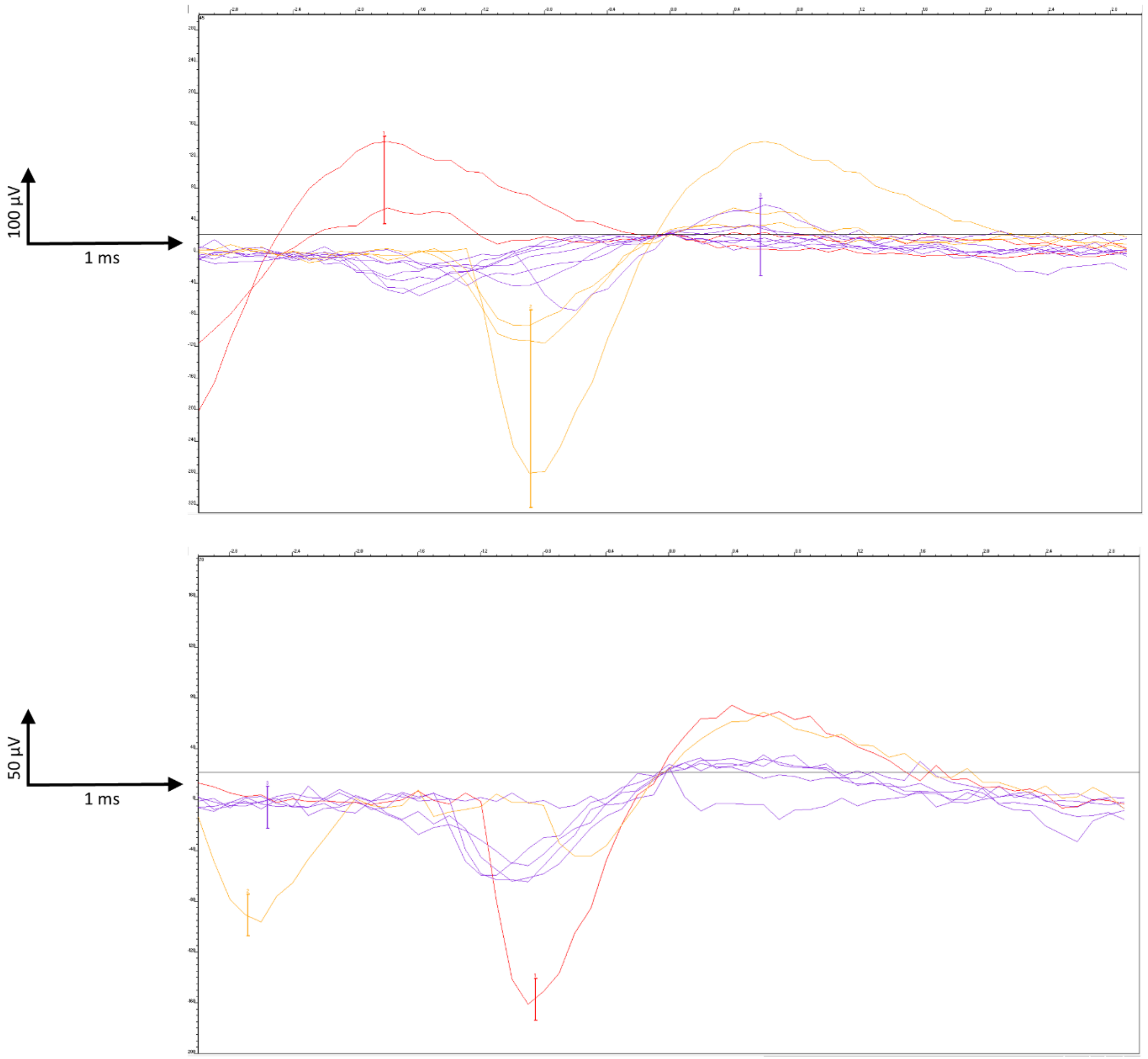

Supplementary Figure S5: MC_Rack electrode recording of 20HE induced RML12 culture spontaneous activity. Each figure corresponds to a screenshot from the activity of one electrode over 150 seconds. The spike sorter parameters are as follow: threshold spike detection = 22 µV, 30_{max} overlay spikes, 3 units of spike sorting (in yellow, purple and red).

SUPPLEMENTARY VIDEOS

Supplementary Video S1. Time lapse video of *Aedes albopictus* RML12 cell line culture, 24 hours post seeding in a well with regular L15 medium (10% FCS).

Supplementary Video S2. Time lapse video of *Aedes aegypti* primary neurons, 24 hours post seeding in a well. The culture shows cell extensions of the neuronal network.

Supplementary Video S3. Time lapse video of *Aedes albopictus* RML12 cell line culture, 24 hours post seeding in a well with serum free L15 medium 20HE supplemented (2 µg/ml)
